# Photo-Catalytic Properties of TiO_2_ Supported on MWCNTs, SBA-15 and Silica-Coated MWCNTs Nanocomposites

**DOI:** 10.1186/s11671-015-1137-3

**Published:** 2015-10-30

**Authors:** Nteseng O. Ramoraswi, Patrick G. Ndungu

**Affiliations:** School of Chemistry, University of KwaZulu-Natal, Westville Campus, Durban, South Africa; Department of Applied Chemistry, University of Johannesburg, P.O. Box 17011, Doornfontein, Johannesburg 2028 South Africa

**Keywords:** Carbon nanotubes, Titanium dioxide, SBA-15, SBA-MWCNT composite, Mesoporous, Photo-catalyst

## Abstract

Mesoporous silica, specifically SBA-15, acid-treated multi-walled carbon nanotubes and a hybrid nanocomposite of SBA-15 coated onto the sidewalls acid-treated multi-walled carbon nanotubes (CNTs) were prepared and used as supports for anatase TiO_2_. Sol-gel methods were adapted for the synthesis of selected supports and for coating the materials with selected wt% loading of titania. Physical and chemical properties of the supports and catalyst composite materials were investigated by powder X-ray diffraction (XRD), Raman spectroscopy, thermogravimetric analysis, scanning electron microscope (SEM), high-resolution transmission electron microscope (HRTEM), UV-vis diffuse reflectance spectroscopy and fluorescence spectroscopy. The photo-activity of the catalyst composites were evaluated on the decolorisation of methylene blue as a model pollutant. Coating CNTs with SBA-15 improved the thermal stability and textural properties of the nanotubes. All supported titania composites had high surface areas (207–301 m^2^/g), altered band gap energies and reduced TiO_2_ crystallite sizes. The TiO_2_/SBA-CNT composite showed enhanced photo-catalytic properties and activity than the TiO_2_/SBA-15 and TiO_2_/CNT composites. In addition, an interesting observation was noted with the TiO_2_/SBA-15 nanocomposites, which had a significantly greater photo-catalytic activity than the TiO_2_/CNT nanocomposites in spite of the high electron-hole recombination phenomena observed with the photoluminescence results. Discussions in terms of morphological, textural and physical-chemical aspects to account for the result are presented.

**Electronic supplementary material:**

The online version of this article (doi:10.1186/s11671-015-1137-3) contains supplementary material, which is available to authorized users.

## Background

Titanium dioxide (TiO_2_) is a stable, well-characterized and inexpensive semiconductor [1]. As one of the most well-studied metal oxides, it has been exploited in the pigmentation, health and catalysis domains amongst others [2]. Despite some of its drawbacks, one interesting and widely used application is as a catalyst in photo-catalytic reactions targeted at removing organic pollutants from water [1–3]. As a photo-catalyst, the main limitation with TiO_2_ is its relatively wide band gap energy, and as a result, the material cannot be effectively used with the visible light energy that filters through to the earth’s surface [2, 4]. To overcome this key disadvantage, there are various strategies that have been employed to reduce the band gap energy and thus improve the generation of charge carriers and at the same time try to increase the lifetime of the charge carriers, decrease the recombination rate of the generated electron and holes, and thus enhance the overall photo-catalytic efficiency of TiO_2_. These methods can include the addition of metals and non-metals either as a dopant or surface deposit and the use of supports, or the TiO_2_ can be carefully synthesised as nanotubes, nanofibers, thin films, nanoparticles or as a mesoporous structure [1–5].

Of particular interest is the use of mesoporous samples which offer the advantage of high specific surface area, nano-sized particles and ordered pores. The significance of these physical properties is that there are a greater number of active sites due to the larger surface area and improved transfer rates of reactants and products as a result of the large and well-organised pore geometry [4–6]. However, with TiO_2_ photo-catalysts, the walls of the mesoporous material must be highly crystalline; otherwise, any amorphous phases will lead to high recombination rates or trapping of the charge carriers, thus decreasing overall photo-catalytic efficiency [5–10].

Another interesting perspective in terms of improving overall photo-catalytic efficiency of TiO_2_ is to use a support such as SBA-15 or carbon nanotubes. SBA-15 is a silica-based material with relatively large pores and thick walls (making it very hydrothermally stable), has both microporosity and mesoporosity, and can be easily synthesised in a relatively short period of time with tunable morphological characteristics. Such desirable features have led to SBA-15 being applied and investigated in a wide range of areas [11]. There have been several studies reported in the literature that use TiO_2_ supported on SBA-15 for photo-catalytic degradation of methylene blue [12–16]. For example, Yang et al. [16] studied various weight percentages of titania dispersed on SBA-15, using a surfactant free sol-gel method, and found that 30 wt% was the optimum photo-catalyst for the degradation of methylene blue. Whereas, Sahu et al. studied the effect of calcination temperature on the photo-catalytic activity of various weight percentages of titania dispersed on SBA-15 and found that 30 wt% samples calcined at 700 °C were most effective for the degradation of methylene blue [15]. Another work by Nava et al. found that a 31 wt% titania on SBA-15 was very effective in the degradation of methylene blue due to high dispersion, surface area, excellent crystallinity, large number of surface hydroxyl groups and superior access to photo-active sites [14]. An interesting study that looked at the sorption properties and photo-catalytic activities of titania on SBA-15 was done by Lachheb et al., and they found that the composites were effective under UV, visible and solar irradiation for the degradation of methylene blue [12].

Carbon nanotubes have been extensively studied and applied in areas such as catalysis, biomedical applications, structural composites, advanced electronic devices, solar cells, batteries and various other functional systems [17, 18]. Recently, Melchionna et al. have provided an extensive review on the role of carbon nanotubes (CNTs) in heterogeneous catalysis [17]. In terms of catalytic supports, key notable features with CNTs are the sp^2^ hybridization of the carbon-carbon bonds and the cylindrical shape of the graphene sheets which provide characteristic and synergistic physical-chemical properties for catalytic processes [17, 18]. Recent R&D has shown that multi-walled carbon nanotubes (MWCNTs) enhance the photo-catalytic activity of TiO_2_ nanoparticles [17, 19–29]. Some of the interesting findings with the earlier work with titania on MWCNTs have shown that excellent physical-chemical contact (as opposed to physical mixing) is key to improved activity; the enhancement is thought to occur via longer lived electron-hole charge carriers that eventually produce greater amounts of radicals, the CNTs provide additional sorption properties, and the composites have superior visible light sorption properties [17, 19–27]. In addition, the use of titania-carbon nanotubes has also shown to create hetero-junctions in the composite and in turn enhance photo-catalytic efficiency [17, 20, 21, 29]. Further improvement of the photo-catalytic properties of TiO_2_/MWCNTs composites with silver nanoparticles via an enhanced plasmonic resonance effect was recently reported by Hintsho et al. [19]. In our current work, we investigate whether the photo-catalytic properties of such composites can be enhanced with a layer of mesoporous silica. The photo-catalytic properties of the titania nanocomposites were tested with methylene blue (MB) as a model pollutant. To the best of our knowledge, titania particles suspended on silica-coated carbon nanotubes composite (TiO_2_/SBA-CNT) have not been widely reported in the open literature.

## Methods

### Materials and Chemicals

Tetraethyl orthosilicate (TEOS, 98 %) and titanium (IV) isopropoxide (TIP, 97 %) were used as silica and titania precursors, respectively. Poly(ethylene glycol)-block-poly(propylene glycol)-block-pole(ethylene glycol) also known as Pluronic P123 and F127 was employed as surfactant and the structure directing agent for the synthesis of mesoporous materials. Methylene blue, nitric acid (HNO_3_, 69 %), hydrochloric acid (HCl, 69 %) and crude ethanol were purchased from Sigma Aldrich, South Africa. MWCNTs (95 wt%, 8–15 nm outer diameter, 10–50 μm and 233 m^2^/g surface area) were purchased from Cheap Tubes Inc (http://www.cheaptubes.com) (Brattleboro). Argon gas that was purchased from Afrox Limited Gas Co. was specified, the ultrasonic probe used was a Hielscher UP400S and the parameters were set at 30 % amplitude and 0.4 duty cycle.

### Treatment of Multi-Walled Carbon Nanotubes

MWCNTs of weight 2.09 and 100.13 g HNO_3_ were mixed in an Erlenmeyer flask and then dispersed using an ultrasonic probe and a time of 20 min. The mixture was then transferred to a round bottom flask and refluxed under air at 80 °C for 4 h. After cooling to room temperature, the CNTs were recovered by filtering (through Whatman filter paper) under vacuum and washed with distilled water until the filtrate had a near to neutral pH. The recovered product was dried in an oven at 100 °C for 18 h and denoted “aCNTs”.

### Synthesis of SBA-15

For the synthesis of SBA-15, a hydrothermal method was adopted from Meynen et al. [11]. Typically, a solution of H_2_O:HCl (130.00 g:20.00 g) and 4.23 g P123 were mixed in a beaker with a magnetic stirrer bar. The mixture was then refluxed at 50 °C (open to air), and then, a dropwise addition of 9.13 g TEOS was made while stirring. After 7 h and 30 min, the mixture was left to stand for an additional 15 h and 30 min at 80 °C. The product was recovered and washed with three portions of 25.00 g of distilled water. The white product was then dried at 100 °C for 3 h and then calcined in a muffle furnace at 550 °C for approximately 6 h (heating rate ~1 °C. min^−1^).

### Functionalization of MWCNTs with SBA-15

In a typical procedure, 0.5 g of the aCNTs was dispersed in the solvent surfactant mixture (130.0 g H_2_O, 20.0 g HCl and 4.23 g P123) with an ultrasonic probe for 20 min. The dispersed mixture of CNTs was then transferred to a two-necked round bottom flask, and while stirring, the mixture was heated to ~45 °C, and then, TEOS was added dropwise. For the 10, 20 and 30 wt% SBA-15 coating on aCNTs, 0.19, 0.44, and 0.79 g of TEOS was added respectively. After stirring, the temperature was increased to 80 °C and the mixture was left standing for 15.5 h. Once cooled to room temperature, the product was recovered through filtration (Whatman filter paper) and washed with three portions of 25.0 g of distilled water. Finally, the product was dried at 100 °C for 18 h and then calcined in a muffle furnace at 400 °C for 6 h (heating rate ~1 °C.min^−1^).

### Synthesis of Mesoporous TiO_2_

Samples were prepared by dissolving 15.01 g of pluronic F127 in 50.00 g ethanol and treating the solution with an ultrasonic probe. Then, 1.26 g TIP was slowly added to the solution and stirred for 30 min. Distilled water (50.00 g) with pH 1.0, adjusted using HCl, was added dropwise to the solution and stirred for a further 1 h before letting the mixture age in air without stirring for 24 h. Ageing was continued in a convection oven set at 100 °C for 6 days. The sample was heat treated under Argon flow at 400 °C for 6 h and then calcined in air at 400 °C for 8 h (both heating rates ~1 °C.min^−1^).

### Preparation of TiO_2_ Composites

Approximately 0.80 g of the support material, either aCNTs, SBA-15 or 30 wt% SBA-CNTs, was added to solutions of ethanol and pluronic F127, prepared as described above. The mixture was then further treated with an ultrasound probe. Then, ~0.44 g TIP was added, and then, the mixture was stirred for 30 min, after which distilled water (acidified) was added as described above. Ageing, heat treatment and calcination steps were the same as those for the unsupported mesoporous TiO_2_. The approximate loading was 10 wt% TiO_2_. Materials were denoted TiO_2_/SBA-15, TiO_2_/CNTs and TiO_2_/SBA-CNT, respectively.

### Material Characterization

Textural properties of materials were studied at 77 K on a Micrometrics Tri-Star II 3030. Samples were prepared by degassing on a Micrometrics Flow Prep (060) under nitrogen flow at 90 °C for 1 h and 200 °C for 4 h before analysis.

The X-ray diffraction (XRD) patterns were recorded on a D8 Advance diffractometer (BRUKER AXS), with Cu-kα radiation of *λ*Kα_1_ = 1.5406 Å using the pore size distribution (PSD) Vantec-1 detectors at a scanning speed of 0.5 s/step. X-ray tube voltage and current were set at 40 kV and 40 mA, respectively.

Morphology studies were observed from images captured on a JEOL (JEM 1010) transmission electron microscope (TEM), a JEOL (JEM 2100, at 200 kV) high-resolution transmission electron microscope (HRTEM) and a LEO 1450 scanning electron microscope (SEM).

Thermal properties of the materials were investigated on a PerkinElmer Simultaneous Thermal Analyzer (STA 6000) using a heating rate of 10 °C.min^−1^ under air flow.

Raman spectroscopy measurements were generated from a DeltaNu Advantage 532™ spectrometer with 1800 lines.mm^−1^ grating.

Photoluminescence (PL) studies were carried on a fluorescence spectrophotometer, LS55, PerkinElmer and a front probe surface accessory LS55 model solid probe. The excitation wavelength was 310 nm.

UV-vis diffuse reflectance spectra were measured on an Ocean Optics spectrometer (high-resolution HR 2000) spectrometer, employing the Ocean Optics Spectrasuite software and a tungsten halogen light source.

### Photo-Catalytic Tests

The photo-catalytic activity was evaluated by measuring the absorbance of 10 mg.L^−1^ MB solution under visible light irradiation in the presence of 0.13 g.L^−1^ catalyst. In a typical experiment, the solution for the photo-catalytic measurements was prepared by adding 20 mg of the respective catalyst to 150 mL aqueous solution of MB (10 mg.L^−1^). Prior to irradiation and measurements, the solution was covered with foil and stirred for 10 min and then stirred in the dark for an hour to attain adsorption equilibrium. The mixture was then exposed to visible light irradiation 32 W (220–240 V/4 U/6400 K) day light halogen lamp while stirring at room temperature. Aliquots of 1.5 mL were sampled over 160 min at 20-min intervals. The aliquot was then centrifuged for 10 min at 140 rate per minute (RPM) × 100. The decolorization/degradation of the dye was evaluated by measuring the absorption of methylene blue solution at the most intense band appearing at 665 nm with a UV-vis spectrophotometer (Biochrom, Libra S6).

## Results and Discussion

### XRD Analysis

**Fig. 1:**
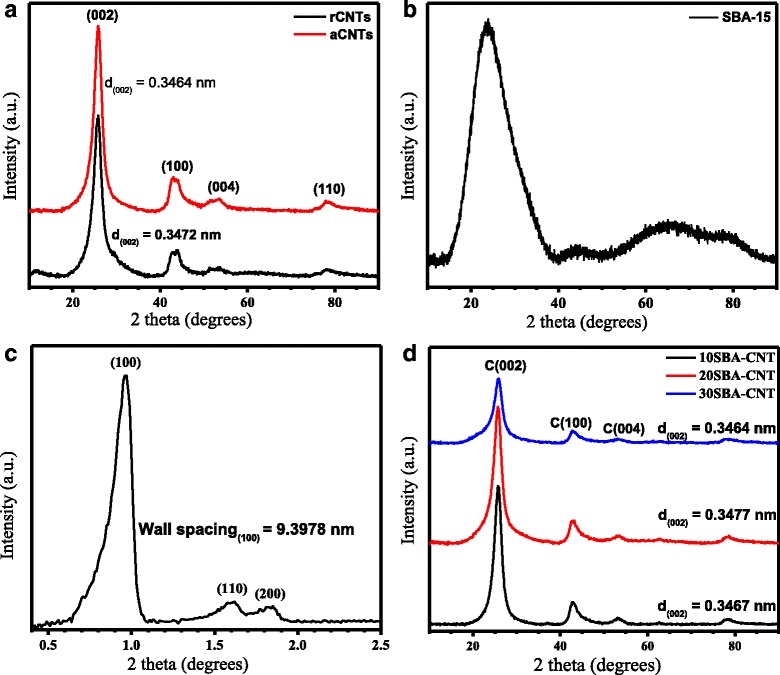
**a** The XRD pattern of raw and acid-treated CNTs. **b** The wide-angle XRD pattern for SBA-15 samples. **c** The low-angle XRD pattern for SBA-15. **d** The wide-angle XRD pattern for various wt% loadings of SBA-15 coated on CNTs

Figure 1a depicts the XRD patterns of the pristine and acid-treated CNT structures. The characteristic peaks of the CNTs appear at 2*θ* values of 26.00° and 43.40°. The strong and sharp diffraction peak at 2*θ* = 25.70 was indexed to the (002) reflection of graphite [30]. The relative sharpness of the peak after acid treatment suggests that the graphitic structure was preserved. The other peaks at 2*θ* = 43.90°, 53.70° and 78.10° correspond to (100), (004) and (110) reflection planes, respectively. The inter-planar spacing (*d*) was calculated using Bragg’s law (*nλ* = 2*d*sin*θ*) with the peak at 2*θ* = 25.70°, and a slight variation was observed between the raw and acid-treated samples. It has been reported that a decrease in the order of crystallinity for CNTs causes the XRD peaks to broaden and shift the (002) reflection plane towards lower angles [31]. Our results are similar to what has been reported previously. In addition, we also observed that functionalising the tubes slightly increased the full width at half maximum (FWHM) from 2.44° with the raw CNTs to 2.47° with the acid-treated CNTs (Table 1). An increase in the FWHM has been reported to be due to long acid treatment times damaging and thus reducing the overall crystallinity of the MWCNTs [31]; however, the slight increase with our results suggests that the treatment protocol removes amorphous carbons and residual catalyst particles without significantly damaging or degrading the overall crystallinity of the MWCNTs.

**Table 1:** Summary of material properties according to the XRD pattern analysis

Support	2*θ* (°)	FWHM (°)	Interplanar spacing *d*_(100)_
rCNTs	25.72	2.44	0.3472^a^
aCNTs	25.66^a^	2.57^a^	0.3464^a^
aCNTs	43.47	2.47	0.2082
10SBA-CNT	43.08	1.77	0.2100
20SBA-CNT	43.11	2.02	0.2098
30SBA-CNT	43.24	2.20	0.2092

^a^ Calculations performed using the (002) reflection plane of CNTs

Figure 1b,c presents the wide and low angle diffraction patterns, respectively, of SBA-15 and suggest that the pores in SBA-15 were highly ordered as deduced from the characteristic diffraction patterns at low angles. The peaks at 2*θ* = 0.94°, 1.62° and 1.85° were indexed to the (100), (110) and (200) planes, which have been reported to correspond to the 2D hexagonal structure of SBA-15 [11]. The wall thickness calculated from the XRD pattern using the equation (Wt = 2*d*_(100)_/3^1/2^ − pore diameter) was found to be 5.70 nm, in agreement with previous reports [11]. A broad peak at 2*θ* = 24° was observed, and it was attributed to the amorphous SiO_2_ character [14, 32].

Introducing SBA-15 to the tube surface resulted in the characteristic peak for the CNTs (at 2*θ* = 25.70°) to gradually decline and the peak to broaden slightly with increasing silica content (Fig. 1d). However, the broad silica peak at 2*θ* = 24° overlaps with the CNT peak at 2*θ* = 25.70°; thus, analysis within that region of the XRD patterns must take the overlap into consideration. Alternatively, analysis of a non-overlapping peak with the CNTs may provide some insight on how the addition of SBA-15 affects the CNTs. Analysis of the C(100) reflection at 43.00° revealed that the FWHM decreased on the addition of silica and then increased with the silica loading (Table 1). However, the interplanar spacing increased on the addition of 10 wt% silica and then decreased slightly with increasing silica loading (Table 1).

FWHM has been shown to decrease as the CNT outer diameter increases [33, 34], and in contrast, the interplanar spacing decreases with increasing diameter of CNTs [33–35]. Functionalisation of the tube wall has also been noted to have an effect on these two parameters [34, 35], and the changes observed with the modified MWCNT samples clearly indicate that the silica has successfully functionalised the nanotubes. Since mesoporous silica undergoes some shrinkage during calcination procedures [36], the changes seen with the XRD parameters may indicate that the silica has uniformly coated the nanotubes and the structural changes during calcination have introduced some additional strain to the MWCNTs and resulted in the changes observed with the XRD parameters. It is interesting to note that the FWHM for the overlapping peak at 2*θ* ~ 26° broadens and follows a similar trend to the non-overlapping peak (2*θ* = 43.00°) for the CNTs.

**Fig. 2:**
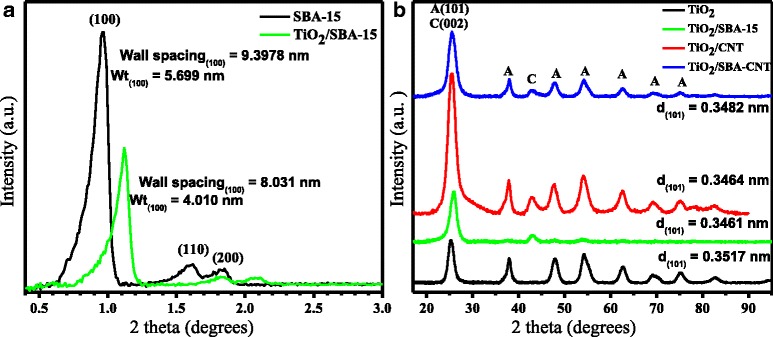
Low-angle XRD pattern of SBA-15 and TiO_2_/SBA-15 nano-composite (**a**) and wide-angle XRD pattern of TiO_2_ composites (**b**), where *A* and *C* denote anatase and carbon nanotube phases, respectively

Figure 2a compares the low-angle X-ray diffraction patterns for SBA-15 and TiO_2_/SBA-15 composites. The low-angle measurements with the CNT composites did not show any distinctive patterns and are not included. The shift of the low-angle peaks and the decrease in intensity with the incorporation of TiO_2_ is a clear indication that the titania has coated the mesopores of the SBA-15 without any significant pore collapse. Similar results have been reported in the literature [15, 37, 38].

The wide-angle XRD patterns for the titania and various samples of supported titania are presented in Fig. 2b. All the samples did show the characteristic peaks for titania in the anatase phase with no evidence of the dominant rutile peak that appears at around 2*θ* values of 27° and 56°. It is interesting to note that the TiO_2_/SBA-15 composite showed broad and low-intensity diffraction peaks of the titania in the anatase phase, and this suggests that the TiO_2_ particles are well dispersed in the SBA-15 framework which is in agreement with the low-angle XRD analysis on the composite.

Typically, the most intense peak for the anatase phase of titania, at 2*θ* ~ 25°, is analysed to determine the crystallite size (using the Scherrer equation). However, the various supports used have definitive XRD peaks within a similar region; thus, Table 2 provides data using the (211) reflection at 2*θ* ~ 54°, which was clearly present with the various samples.

**Table 2:** Properties of TiO_2_ catalyst on supports according to XRD pattern analysis using the (211) reflection peaks of anatase TiO_2_

Catalyst	2*θ* (°)	FWHM (°)	Crystal size (nm)
TiO_2_	54.43	1.91	4.69
TiO_2_/SBA-15	54.04	1.97	4.53
TiO_2_/ CNT	54.18	1.99	4.48
TiO_2_/SBA-CNT	54.30	1.84	5.86

From the data in Table 2, there are some small differences with the titania crystallite size when the materials are placed on the different supports. The slight shift to lower angles from unsupported to supported titania and changes with the FWHM correlate with the change in crystallite size. Overall, the SBA-15 and CNT supports result in smaller titania crystallites, while the nanocomposite support of SBA-15/CNT results in slightly larger titania crystallites. The effect that this has on the photo-catalytic properties is discussed below.

### Raman Spectroscopic Analysis

Raman spectra of rCNTs and aCNTs (not shown) displayed the characteristic D and G bands at approximately 1350 and 1580 cm^−1^, respectively. The D band is associated with disorder (sp^3^) carbons and the G band with the crystalline graphite structures (sp^2^ carbons) of CNTs [21, 39, 40]. The integrated area of the D and G band intensity ratio (*I*_D_/*I*_G_) for rCNTs and aCNTs were 1.37 and 1.11, respectively. The decrease shows that there was an increase in the graphitic nature of the tubes due to the removal of amorphous carbon that may have been on the tube walls or the removal of separate particles of amorphous carbon [21, 40, 41]. This result is line with the XRD analysis that showed that there were more graphitic carbon structures after acid treatment. Gerber et al. [42] observed similar phenomena of an increasing *I*_D_/*I*_G_ ratio and cleaner surface morphology for acid-treated tubes. The FWHM of the bands in acid-treated CNTs was narrower than those in raw CNTs (Table 3). The decrease is associated with increased crystallinity within the aCNTs [43].

**Table 3:** Comparison of the Raman spectra parameters on SBA-CNTs composites

Sample	FWHM		Ratio (*I*_D_/*I*_G_)
	D band ≈1350 (cm^−1^)	G band ≈1577 (cm^−1^)	
rCNTs	74.85	85.16	1.37
aCNTs	70.14	76.52	1.11
10SBA-CNT	65.92	81.70	1.46
20SBA-CNT	66.00	76.90	1.21
30SBA-CNT	35.09	78.55	1.42

Raman spectra of the SBA-15 samples (see Additional file 1) exhibited spectroscopic features typical of amorphous SiO_2_. The bands observed at 410 and 813 cm^−1^ were assigned to siloxane linkage (Si-O-Si) and bands at 493 and 654 cm^−1^ were assigned to the four- and threefold membered siloxane rings, respectively. The very intense band at approximately 1134 cm^−1^ was related to surface silone (O_3_Si-OH) stretching vibrations [44, 45]. These features were still evident in the SBA-CNT composites (see Additional file 1) which also had the characteristic Raman peaks for CNTs. The most dominant peak for the SBA-15 shifted to 1050 cm^−1^, and this variation does indicate a covalent attachment between the CNTs and SiO_2_ [46]. The *I*_D_/*I*_G_ ratio of the SBA-CNT composites increased with the coating of SBA-15 (Table 3). Such a result could have been due to the acidic solution used to coat the SBA-15 onto the nanotubes, functionalising the sidewall of the CNTs and thus increasing the disorder with the CNTs. However, from Table 3, the FWHM of the D-band decreases and such a decrease is likely due to the covalent attachment of the silica to the defect sites on the CNT sidewalls, and this would account for any effects that would lead to suppression of the D-band vibrations. There is no clear trend with the increase in wt% of SBA-15 on the CNTs and this can be attributed to such factors as difference in nanotube diameters, lengths and degree of functionalization within the samples; a similar observation was made with CNTs subjected to different and sequential oxidation treatments [40]. However, the large decrease in the FWHM of the D-band with the 30 SBA-CNT samples could be due to a greater ratio of TEOS: surfactant allowing for a greater amount of silica to bind the defect sites of the CNTs despite the amount of surfactant used.

The Raman spectra of titania (see Additional file 1) depict vibrational bands at 400, 521 and 644 cm^−1^ which correspond to the anatase modes of B_1g_, A_1g_ overlapping with B_1g_, and E_g_ symmetry, respectively. These modes are associated with anatase phase titania [14], and the analysis is in agreement with the identified phase from the XRD pattern. With the TiO_2_ on the various supports, the characteristic peaks for the anatase phase were highly suppressed, and this can be attributed to the highly dispersed and small particles of titania on the supports (which correlates with the HRTEM analysis); similar results have been reported in the literature [14]. The characteristic peaks corresponding to the supports were clearly evident, with the CNTs containing composites depicting the well-defined D and G bands of the nanotubes at ≈1350 and 1600 cm^−1^, respectively.

### TGA Studies

**Fig. 3:**
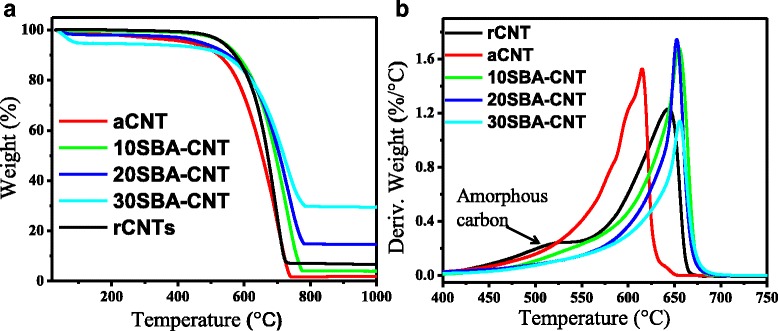
Thermogravimetric pattern (**a**) and derivative weight pattern (**b**) of CNTs and CNT-SBA-15 composites

Thermograms presented in Fig. 3a showed that the decomposition onset temperature for the acid-treated CNTs was lower than that of raw CNTs. This could be as a result of the presence of highly oxidative functional groups in aCNTs which are prone to oxidation at low temperatures [31]. The decomposition feature of the rCNTs at ≈400 °C suggests that the amount of impurities, either in the form of amorphous carbons, were relatively high (≈7.1 wt%). Previous reports in the literature have shown that amorphous carbon decomposes at lower temperature than carbon nanotubes [40, 47]. Thermal stability of materials was deduced from the derivative weight vs. temperature curves, and the oxidation temperatures shifted from 643 to 614 °C for rCNT and aCNTs, respectively. After purification, the shoulder peak at 400 °C had reduced somewhat and is a qualitative indication that there were reduced amounts of amorphous carbon in the aCNTs. Additionally, the aCNTs depicted a narrower oxidation peak, which indicated high purity with the tubes and a more uniform sample [48]. The decrease in residue in the acid-treated CNTs (≈2.1 wt%) as compared to raw CNTs (≈7.1 wt%) does show that a significant amount of the metal catalyst was removed by the treatment procedure.

Overall, the acid treatment process did introduce oxygen functional groups onto the surface of the CNTs (see Additional file 1), such as carbonyl groups associated with ketone, carboxylic or quinone groups (1638 and 1618 cm^−1^) and characteristic OH moieties (3474 and 3415 cm^−1^) from surface sorbed water or COOH groups [49].

The decomposition behaviour of the composites showed that as the SBA-15 content was increased, the residual mass with the samples changed from ≈7, 17 and 29 wt%, respectively, for 10, 20 and 30 wt% SBA-CNT composites. This could indicate that at low silica loadings, there is some loss of silica during the coating procedure, possibly to the reaction vessel, or the treatment time may need some adjustment to ensure more complete condensation on the CNTs. However, one interesting aspect from the TGA analysis was the confirmation that the silica coats the CNTs at all weight loadings, since the maximum rate of oxidation was observed at 658, 656 and 659 °C for the 10–30 wt% SBA-CNT samples (aCNTs ~614 °C). A similar phenomenon was observed by Paula et al. [46], and they attributed the trend to the deposition of silica onto the graphitic sheets of the tubes.

### Morphological Analysis

**Fig. 4:**
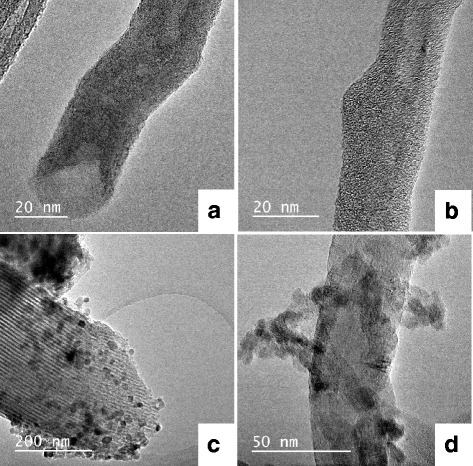
Selected HRTEM micrographs of **a** acid-treated MWCNTs (aCNTs) showing open tube ends, **b** 30 wt% SBA-15 on MWCNTs (30 SBA-CNT), **c** 10 wt% titania on SBA-15 and **d** is the 10 wt% TiO_2_ on the SBA-CNT nanocomposite

Figure 4 presents HRTEM micrographs for selected samples. The MWCNTs before treatment (not shown) did have a significant amount of nanotubes with closed ends, and after acid treatment (Fig. 4a), the nanotubes were typically observed with open ends and somewhat smoother tube walls. The increase in tube openings will result in a decrease in thermal stability of the aCNTs, as observed with the TGA results, and the smoother walls will show an improvement in the crystallinity as determined by the Raman analysis.

Figure 4b is an image of the sample with 30 wt% SBA-15 on the MWCNTs. The silica coating displays a wormlike structure, and this can be attributed to the change in the meso-structuring of the soft template due to the presence of CNTs during the synthesis procedure. In a study by Paula et al. [46], the authors showed that increasing the concentration of the soft template/surfactant can improve the pore ordering and surface area of silica/CNT nanocomposites, and use of initial ratios for the synthesis of mesoporous silica can result in relatively poor mesophases when CNTs are added. Our results agree with these earlier observations. Further analysis on the nanocomposites was done by examining over 50 coated nanotubes per sample and measuring the thickness of SBA-15 on the MWCNTs. The average thickness of the SBA-15 coating was found to be 3.9, 4.5 and 4.8 nm for the 10, 20 and 30 wt% SBA-CNT composites, respectively. At 30 wt% loadings, it was noted that individual tubes were uniformly coated with SBA-15 unlike those for the 10 and 20 wt% composite materials. This variation in the coating may account for the lack of a trend observed with the Raman *I*_D_/*I*_G_ ratio of the SBA-CNT samples, where the 20 wt% composite had an *I*_D_/*I*_G_ value of 1.21 (see Table 3, 20SBA-CNT) and that of the 10 and 30 wt% samples were 1.46 and 1.42, respectively (see Table 3, 10SBA-CNT and 30SBA-CNT).

The unsupported mesoporous TiO_2_ did not show any clear or regular porosity (not shown) when examined with the HRTEM; however, the SBA-15 with (Fig. 4c) and without (not shown) TiO_2_ did display the characteristic porous structure expected for SBA-15. In addition, Fig. 4c shows that the TiO_2_ nanoparticles were dispersed on the SBA-15 support. The XRD patterns for the TiO_2_ on SBA-15 showed relatively low intensity and broad peaks which can be attributed to the small well-dispersed nanoparticles observed with the HRTEM analysis. The introduction of TiO_2_ did not alter the regular, long range ordered pore structure of SBA-15. Similar morphological studies have been reported for TiO_2_/SBA-15 composites [50].

Micrographs of TiO_2_ on CNTs (see Additional file 1) showed good dispersion and small agglomerates of TiO_2_ particles on the de-bundled acid-treated nanotubes. The observed morphology could have been facilitated by the functional groups along the CNT walls. In contrast, on the SBA-15-coated CNTs, the titania did show distinct agglomeration on the nanocomposite (Fig. 4d).

### Nitrogen Sorption Studies

**Fig. 5:**
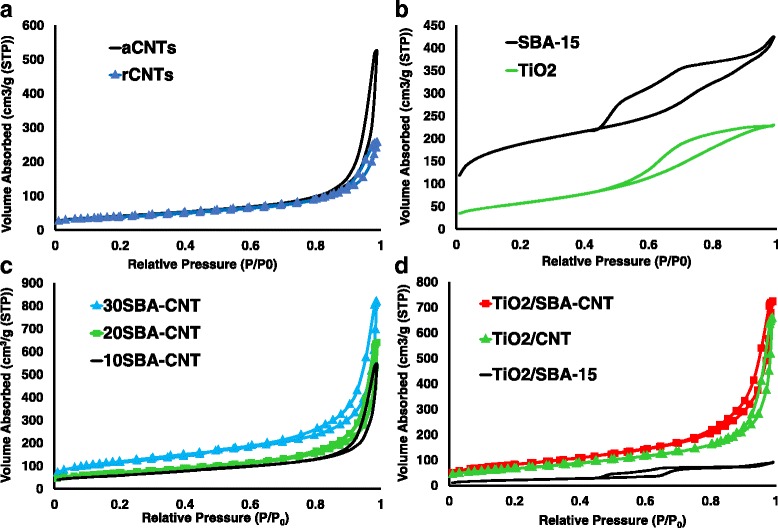
Nitrogen sorption isotherms for **a** raw and acid-treated MWCNTs, **b** SBA-15 and mesoporous titania, **c** the 10–30 wt% SBA-15 on MWCNT samples, and **d** 10 wt% TiO_2_ on MWCNTs, SBA-15 and 30 wt% SBA-15 coated on MWCNT

Figure 5a presents the isotherms for the rCNT and aCNT samples. The samples displayed a type IV isotherm, typical for CNTs [51, 52], and the aCNTs showed an increase in volume sorbed with a slightly wider hysteresis loop at higher *P*/*P*_0_ values. In addition, the PSD changed from the rCNT to aCNT samples (see Additional file 1), with the aCNTs showing greater contribution of porosity in the macroporous regions. This is indicative of de-bundling of the CNTs, and opening of the tube ends during treatment [22], and is in line with HRTEM results presented in Fig. 4a. Figure 5b shows the isotherms for the SBA-15 and titania samples prepared; both show a type IV isotherm indicative of mesoporous materials [11]. The hysteresis loop, in the sorption isotherms, at relative pressures 0.5 <*P*/*P*_0_ <1.0 represents the spontaneous filling of mesopores due to capillary condensation [14], and this is indicative of ordered mesoporous structure with the SBA-15 and titania samples. For SBA-15, the isotherms are in agreement with the micrograph and XRD analysis, and the PSD (see Additional file 1) confirmed that the material had a narrow pore distribution centred at 4 nm.

The nitrogen sorption isotherms at 77 K for the SBA-15-coated CNTs are presented in Fig. 5c. The composites sorbed a greater volume of nitrogen than the individual materials, especially at 20 and 30 wt% SBA-15 loading; this would indicate that the nanocomposite is not simply a mixture of SBA-15 and aCNTs since such a mixture would have values between those of SBA-15 and a CNTs and not the larger values observed for the volume of nitrogen sorbed. Thus, this result highlights that the samples are nanocomposite with a mesoporous silica layer coated along the CNT walls, as per HRTEM observations (Fig. 4b). It is also interesting to note that the composite with 30 wt% loadings of silica had a hysteresis loop that extended over a wider relative pressure range (0.6–1.0 *P*/*P*_0_) than other composites and is attributed to the capillary condensation of gases within the mesopores of the silica that is coated along the CNT walls. The corresponding PSD pattern for the silica nanocomposites (see Additional file 1) showed that the materials pose well-defined mesoporous peaks at ~ 2.6 and 3.5 nm for the 10SBA-CNT and 20SBA-CNT samples, while the 30SBA-CNT sample had a sharp peak at ~4.0 nm. Previous reports in the literature have reported on similar composites with PSD around 2 nm [53–55] and 4 nm [56, 57]. In the current work, the aCNTs displayed similar peaks at ~2 and 3 nm on the PSD graph, and thus, their appearance in the 10 and 20 wt% nanocomposites is due to the low loadings of SBA-15 and the overall preservation of the CNTs textural properties. While the 30 wt% loading having the ~4.0 nm peak in the PSD chart does show that at the higher loadings, the desired high surface area and mesoporosity of the SBA-15 is attained with nanocomposites.

Figure 5d presents the isotherms for the titania supported on SBA-15, aCNTs and 30 wt% SBA-15 on CNTs. The largest change observed with the isotherms was with the titania on SBA-15, and this is attributed to the titania blocking a significant amount of the SBA-15 pore openings.

Textural properties, specific surface area and the titania loading are presented in Table 4. Acid treatment of CNTs increased the surface area of the tubes by ≈9 m^2^.g^−1^. The increase of surface area was expected since TGA, XRD and Raman analysis suggested the removal of amorphous carbons and introduction of various functional groups plus de-bundling of the tubes. The surface area of the SBA-CNTs nanocomposites increased with silica coating thickness on the nanotubes. This was because SBA-15 has very high surface area ranging from 500 to 1000 m^2^/g [11]; thus, its presence on any structure should enhance the surface area to noticeable degrees. The increased pore volumes (0.56, 0.69 and 0.93 cm^3^.g^−1^) in the composites were further evidence that the amount of SBA-15 was increased within the nanocomposite as the loading was increased.

**Table 4:** Titania loading and the textural properties of the CNTs before after acid treatment, the SBA-15, the 10, 20 and 30 wt% SBA-15 coated on aCNTs, the mesoporous titania synthesised in house and the titania supported on the various supports

Property	rCNTs	aCNTs	SBA-15	10SBA-CNT	20SBA-CNT	30SBA-CNT	TiO_2_	TiO_2_/CNT	TiO_2_/SBA-15	TiO_2_/SBA-CNT
*S*_BET_ (m^2^/g)	140.3	149.1	661.6	209.9	256.4	431.41	207.8	241.3	214.0	301.4
*V*_total_ (cm^3^/g)^a^	0.34	0.56	0.63	0.56	0.69	0.93	0.35	0.70	0.43	0.89
PSD (nm) ^b^	2.89	2.89	4.00	3.45	3.45	3.94	3.52	3.29	3.29	5.29
TiO_2_ loading (wt%)	N/A	N/A	N/A	N/A	N/A	N/A	100	13.6	7.1	11.5

^a^ Total pore volume adsorbed at a relative pressures for N_2_ at −196 °C

^b^ Pore size distribution

The specific surface area of the TiO_2_ composites increased when compared to the specific surface area of TiO_2_ nanoparticle, indicative of the dispersion of the titania on the nanocomposites. The loading of titania on the supports (see Table 4) was determined using ICP-OES, and the wt% was a bit higher than expected for the CNT and SBA-CNT supports and lower for the SBA-15 support. The lower value with SBA-15 can be due to the titania depositing within the pores of the SBA-15 and the digestion process being unable to dissolve the titania trapped within the pores. This correlates with Fig. 5d and the notion that the titania blocked the SBA-15 pores.

### UV-vis Analysis on the TiO_2_ and TiO_2_ Nanocomposites

**Fig. 6:**
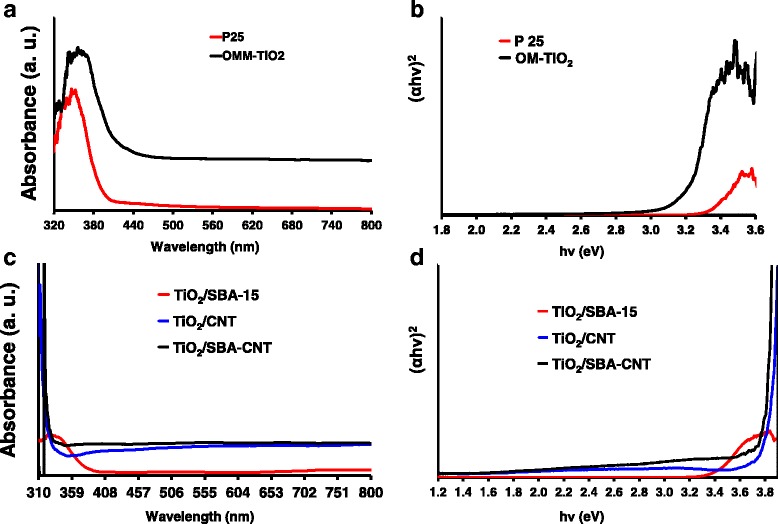
UV-vis diffuse reflectance measurements on P25 and mesoporous titania (**a**) and the corresponding plot of (*αhν*)^2^ vs. *hν* (**b**). **c** UV-vis diffuse reflectance measurements on titania supported on SBA-15, CNTs and SBA-CNT, with **d** showing the corresponding plots of (*αhν*)^2^ vs. *hν*

UV-vis diffuse reflectance measurements and the corresponding plots of (*αhν*)^2^ vs. *hν* of the titania and supported titania samples are presented in Fig. 6a–d, respectively. All of the materials had intense absorptions in the UV-vis range, with a noticeable red shift with the ordered mesoporous titania samples. The band gap can be estimated from the plots of (*αhν*)^2^ vs. *hν* [18, 50, 58, 59], and the estimated band gap energies were 3.18, 3.34 and 3.42 eV for mesoporous the mesoporous titania, P25 sample and the titania supported on SBA-15, respectively. The titania supported on CNTs and SBA-CNT nanocomposites had an extended absorption feature well into the visible end of the spectrum and the corresponding plots of (αhν)^2^ vs. hν had a continuous energy feature which may indicate excitations at low band gap energies. The red shift indicates a decrease in band gap energy and that these materials are active in the visible region of the electromagnetic spectrum.

Titania supported on CNTs have been shown to have an extended absorption feature into the visible range of the electromagnetic spectrum [19, 60–62]. The interesting result here is that the coating of silica does not interfere with the synergistic optical effect seen between CNTs and the titania.

### PL Studies

**Fig. 7:**
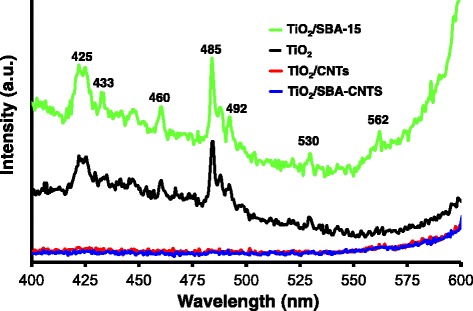
Photoluminescence spectra of TiO_2_ nanoparticles and 10 wt% TiO_2_ on CNTs, SBA-15 and the SBA-CNT nanocomposite. The spectra were captured using an excitation wavelength of 310 nm

PL spectra can be used to probe the defects or structure within nanomaterials [58, 63], and with titania, it has been used to correlate changes with defects due to Ti^3+^ [64]. Figure 7 compares the PL spectra for titania and supported titania samples. The unsupported TiO_2_ and TiO_2_/SBA-15 samples showed peaks at 425, 433, 460, 485, 488, 492 and 530 and a peak at 562 nm which was absent on the unsupported titania. The origin of these peaks are still an intense area of debate and study within the literature, since the PL spectra can originate from defects on the surface of titania [59, 63–65], oxygen vacancies or under-coordinated Ti^3+^ [59, 63, 65], or from self-trapped excitons [59, 63]. In addition, the surface chemistry can affect the PL spectra [65, 66].

The PL emission features between 425 and 433 nm can be ascribed to self-trapped excitons [59]. The peaks between 461 and 492 nm are most likely due to defects and oxygen vacancies within the titania crystallites [59, 63], and the peak at 530 nm is probably due to surface dangling bonds or oxygen vacancies at or near the surface of the titania crystallites [58, 63, 64]. PL spectra for the unsupported TiO_2_ and TiO_2_/SBA-15 were similar, with only a small difference in intensities where the TiO_2_/SBA-15 samples were slightly higher than the unsupported titania. In contrast, a dramatic decrease in the electron-hole recombination rate was observed with TiO_2_/CNT and TiO_2_/SBA-CNT nanocomposites since there were no characteristic peaks observed with the PL spectra. Nanotubes are known to be excellent electrical and thermal conductors; thus, the suppression of the recombination rate is due to the CNTs acting as excellent and effective electron acceptors and transporters, thus preventing the recombination of electrons with the holes. Similar observations have been made in the literature [19, 25]; however, what is interesting to note is that the titania on the SBA-CNT nanocomposite has a very similar suppression effect on the electron-hole recombination rate when compared to the titania on the CNTs. Thus, despite the silica coating, there is still effective generation and transport of generated electrons from the titania nanoparticles to the CNTs. This observation merits further study, but one possible reason is that the thin layer of silica (4.8 nm) on the nanotubes does not act as an effective insulator, or there is some kind of resonance energy transfer mechanism between the titania and the CNTs. Such mechanisms have been observed between titania and silver nanoparticles with a 10-nm silica layer between the titania and the silver [67].

### Photo-Catalytic Studies

MB is a cationic dye, and it has been widely utilised in photo-catalytic reactions as a probe or model system to ascertain the effectiveness of various photo-active nanocomposites [19, 24, 25, 68]. The organic compound, MB, has a characteristic absorption spectrum in the visible part of the electromagnetic spectrum, making it very easy to follow using a suitable spectrophotometer, and the mechanism for its degradation by titania-based materials has been extensively studied [12, 14, 25, 69, 70].

**Fig. 8:**
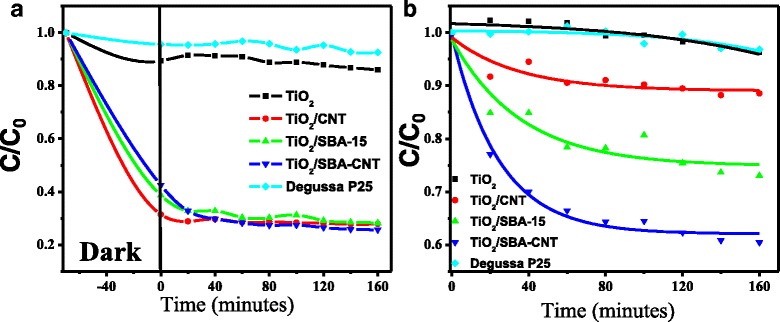
Decolourization of MB in the presence of 30 W halogen lamp and the various TiO_2_ catalysts, sorption equilibrium properties of the materials (**a**) and the photo-catalytic activity (**b**)

Figure 8a compares the decolorisation of MB by titania, P25, 10 wt% titania on CNTs, SBA-15 and SBA-15-CNT nanocomposite. The titania and P25 were ineffective with the low-power light source used, and only absorbed a minimal amount of dye in the dark, and then desorbed some dye on excitation by the light source, with eventually a small amount of the dye being degraded. The supported titania composites effectively absorbed a significant amount of the dye under dark conditions, and when normalising the concentration after absorption in the dark, these composites effectively degraded the dye, as shown in Fig. 8b. Prior work in the literature has shown that MB reaches an absorption-desorption equilibrium in the dark without undergoing any degradation on titania catalysts supported SBA-15 and CNT [12–16, 19, 24–27, 68]. The noteworthy comparison in Fig. 8a is that on illumination, there is a slight increase in MB with the homemade mesoporous titania sample and the P25, which indicates some desorption of MB before the minimal degradation. Whereas, the supported titania catalysts undergo a further drop on illumination, with no slight increase as observed with the P25 and mesoporous titania controls, indicating that the MB absorbed onto the surface of these supported catalysts undergoes degradation rapidly under illumination.

From Fig. 8b, the titania supported on the SBA-15-CNT nanocomposite is the most effective catalyst using the low-power light source. The kinetics and mechanism for the degradation of methylene blue by titania-based photo-catalysts has been well studied in the literature, and the data can be fitted using a pseudo first-order kinetic equations [14, 19, 25, 69–73] and normalising with the surface area of the catalysts used [14, 19, 71]. The results of our analysis are presented in Table 5. What is interesting to note is that the rate constant for titania on SBA-15 nanocomposites is ~37 % greater than the titania on CNTs, and when normalised for surface area, the rate constant is ~44 % greater. Recently, several authors have noted that the intimate contact between titania and CNTs allows for effective separation of charge carriers in the titania/CNT nanocomposites, greatly reduces charge carrier recombination, increases the lifetime of the electron and holes and results in effective and possibly much more efficient pathway for the generation of radicals for the photo-degradation of methylene blue [19, 24, 25]. Thus, from the data on the optical properties presented in Fig. 6 and the PL studies presented in Fig. 7, it would be expected that the titania/CNT nanocomposites would be much more effective than the titania/SBA-15 nanocomposites. Thus, the greater activity of the titania/SBA-15 vs. the titania/CNT nanocomposite is an unexpected result. In addition, the sorption of the dye onto the titania CNT composite was greater than that on the titania on SBA-15. This correlates with the textural characteristics summarised in Table 4, which show that the specific surface area and the total pore volume for the titania on SBA-15 was less than that for the titania on the CNTs; thus, mass transfer effects, differences in surface area or dispersion of the titania may not play a prominent role in terms of accounting for the unexpected differences in activities.

**Table 5:** Pseudo first-order rate constants and the normalised rate constants for the active photo-catalysts

Photo-catalyst	*K* (min^−1^)	*K* (min^−1^ m^−2^photo-catalysts)
TiO_2_/SBA-15-CNT	−2.06 × 10^−03^	−3.41 × 10^−04^
TiO_2_/SBA-15	−1.26 × 10^−03^	−2.93 × 10^−04^
TiO_2_/CNT	−7.95 × 10^−04^	−1.65 × 10^−04^

Hintsho et al. [19] demonstrated that acid-treated CNTs as supports for titania can reduce the photo-catalytic properties of titania supported on CNT nanocomposites. The current work supports these earlier observations and at the same time demonstrates that the introduction of surface sites for the coating of inorganic materials although beneficial for de-bundling and anchoring certain moieties can hamper desired traits.

Coating silica onto MWCNTs has been shown to occur via a co-operative self-assembly mechanism that involves the formation of Si-O-C bonds, especially those located at defect sites along the walls of the CNTs [46]. The greater activity with the SBA/CNT support can be due to the fact that the silica coating is more than likely initiated at the introduced defect sites (see Raman results) on the CNTs, and after coating, these sites are no longer as active as the aCNTs alone; thus, besides the excellent textural properties with the SBA/CNT composites, the high activity is also due to suppression of such defect sites and enhanced generation and separation of the electron-holes and eventual production of the active species for degradation of the dyes.

Another key effect could be due to the optical properties of SBA-15. Mesoporous silica films have been shown to have excellent scattering properties [54] and excellent antireflective properties when coated onto a substrate [55]. The CNTs have excellent optical absorption properties in the UV-vis region [74–76], and thus, any incident light on the CNTs will not reflect or scatter to possibly interact with the dispersed titania, and thus, those photons will not contribute to the generation of electron or holes. However, with the SBA-15, the light can be reflected, scattered or refracted within the mesoporous structure and in doing so may interact with the dispersed titania. In such a scenario, any photons that initially did not interact with the titania may contribute to the generation of electron and holes after scattering by the SBA-15 support and interacting with the dispersed titania. Thus, when comparing titania on CNTs and SBA-15, the scattering phenomena plays a prominent role in a greater rate of electron-hole generation and thus can account for the greater catalytic activity with titania on SBA-15 when compared to titania on CNTs.

In terms of the catalytic activity of the titania on SBA-based supports, the pseudo rate constant is 39 % greater with titania on SBA-CNT vs. titania on SBA-15; however, the normalised surface area rate constant is only ~14 % greater. This may be indicative of the optical absorption properties of the CNTs counteracting the scattering properties of the silica coating; however, it does show that these silica-CNT composite may have tailorable optical-electronic properties via adjusting the thickness and morphology of the silica coating. The combination of SBA-15 and CNTs provides an excellent support with synergistic physical-chemical properties that improve the photo-catalytic properties of titania. Thus, the observed improvement with the SBA-15/CNT nanocomposite is due to several factors, the excellent textural properties, the blocking of defect sites on the aCNT walls, the scattering properties of the SBA-15 and the effective separation of charge carriers by the CNTs.

## Conclusions

Multi-walled carbon nanotubes were successfully functionalised and coated with thin film silica using the sol-gel method. Physical and chemical properties of the materials revealed that silica enhances the textural properties of CNTs at high loadings (30 wt% SBA-15). The use of CNTs, SBA-15 and SBA-CNT composites to support TiO_2_ particles resulted in relatively small titania crystallites, with excellent crystallinity, surface area and porosity. The optical properties of the supported titania nanocomposites showed that the absorption of visible lights was improved on CNTs and SBA-CNT supports, and the electron-hole recombination rate was significantly decreased on the CNT-based materials and only slightly on SBA-13 supports. Photo-catalytic tests on TiO_2_, TiO_2_/SBA-15, TiO_2_/CNTs and TiO_2_/SBA-CNT catalyst composites revealed that the supported catalysts were very effective in the decolorization of MB when compared to P25 and an in-house sample of mesoporous titania. When comparing SBA-15, CNTs and SBA-CNT supports, an interesting outcome was that the SBA-15 support was more efficient than the CNT support despite having a higher electron-hole recombination rate than the titania supported on CNTs. This is most likely due to the excellent scattering properties with SBA-15 allowing for a much higher rate of electron-hole generation that is significantly greater than the recombination rate and thus allowing for more active species being produced to decolourise the MB. This effect, along with the excellent textural properties with the SBA-15 coated on the CNTs, synergistically produce a superior support when compared to CNTs or SBA-15 alone. Thus, the method developed produces an excellent nanocomposite of SBA-15 coated onto CNTs that enhances the textural properties of the CNTs, maintains and takes advantage of the CNTs excellent electronic and opto-electronic properties and opens up the possibility of using such materials in a diverse range of photo- or electrochemical energy conversion systems.

## Additional file

Additional file 1:
**Supplementary Information.** This file contains Figures S1–S7.
