# Supplementary material for: Photo-Catalytic Properties of TiO2 Supported on MWCNTs, SBA-15 and Silica-Coated MWCNTs Nanocomposites
Source: Nanoscale Res Lett. 2015 Oct 30;10:427. doi: 10.1186/s11671-015-1137-3 (PMC4627977; doi:10.1186/s11671-015-1137-3)
Supplement: Additional file 1: — Supplementary Information. This file contains Figures S1–S7. [file 11671_2015_1137_MOESM1_ESM.docx]

***Photo-catalytic Properties of Anatase TiO_2_ supported on CNTs, SBA-15 and silica coated MWCNTs nano-hybrid***

Nteseng O Ramoraswi^a^ and Patrick G Ndungu^b^

*^a^School of Chemistry, University of KwaZulu-Natal, Westville Campus, Durban, South Africa*

*^b^Department of Applied Chemistry, University of Johannesburg, P.O. Box 17011, Doornfontein, Johannesburg, 2028, South Africa*

**Supplementary Information**

**S1:** Raman spectra of SBA-15.

**S2:** Raman spectra of SBA-15 coated aCNTs.

**S3:** Raman spectra of the synthesized TiO_2_.

S4: Raman spectra of TiO_2_ on different supports.

**S5**: FTIR spectra of raw (a) and functionalized (b) CNTs


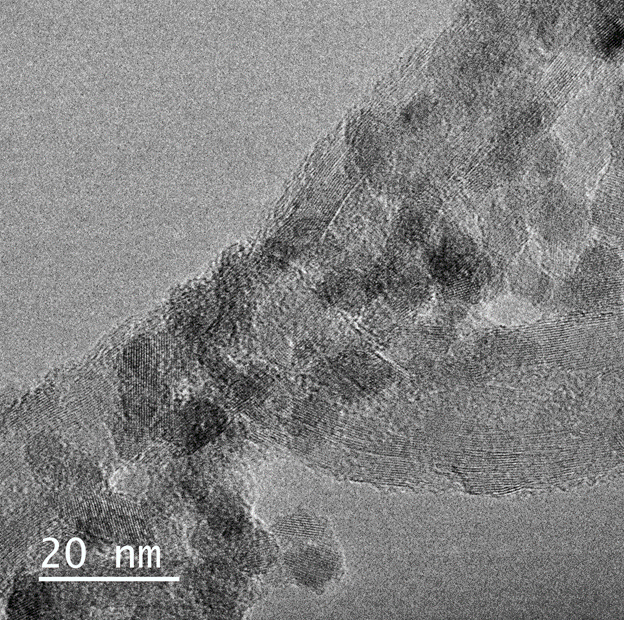


S6: HRTEM micrograph of TiO_2_/CNTs.

**S7:** Pore size distribution for the raw and acid treated CNTs (a), the SBA-CNT nanocomposites and SBA-15 (b), and the unsupported and supported titania materials (c).
